# Snake Venom Makeover: Age-Dependent Variations in Procoagulant Biochemistry of Egyptian Saw-Scaled Viper (*Echis pyramidum pyramidum*) Venom

**DOI:** 10.3390/toxins17030149

**Published:** 2025-03-19

**Authors:** Alex Barker, Lee Jones, Lachlan A. Bourke, Lorenzo Seneci, Abhinandan Chowdhury, Aude Violette, Rudy Fourmy, Raul Soria, Matt Aldridge, Bryan G. Fry

**Affiliations:** 1 Adaptive Biotoxicology Lab, School of the Environment, University of Queensland, St Lucia, QLD 4072, Australia; alex.barker@student.uq.edu.au (A.B.); lee.jones@uq.edu.au (L.J.); l.bourke@uq.net.au (L.A.B.); uqlsenec@uq.edu.au (L.S.); abhinandan.choudhury@uq.edu.au (A.C.); 2 Alphabiotoxine Laboratory srl, Barberie 15, 7911 Montroeul-au-bois, Belgium; aude.violette@alphabiotoxine.com (A.V.); info@alphabiotoxine.com (R.F.); 3 Inosan Biopharma, 28001 Madrid, Spain; rsoria@inosanbiopharma.com; 4 MicroPharm Limited, Newcastle Emlyn SA38 9BY, UK; matt.aldridge@micropharm.co.uk

**Keywords:** *Echis*, coagulopathy, antivenom, factor activation

## Abstract

*Echis* species (saw-scaled vipers) are WHO Category 1 medically significant venomous snakes with potent procoagulant venoms, which cause lethal venom-induced consumptive coagulopathy in human victims. Despite clinical presentations of bites varying significantly between individuals within the same species, the contribution of age-related changes in the venom biochemistry has not been investigated. This study investigated the ontogenetic changes in *Echis pyramidum pyramidum* venom and its impact on therapeutic efficacy. The efficacy of various antivenoms (Echitab, Echitab+ ICP, Inosan MENA, Inosan Pan African, and SAVP-*Echis*) was tested against both venom phenotypes. While both neonate and adult venoms were procoagulant, there were differences in the underlying biochemistry. Neonate venom was found to potently pathophysiologically activate Factor VII and Factor X, and to a lesser degree Factor XII. In contrast, adult venom was a slower clotter, less potent in activating FVII, equipotent with neonate venom on FXII, and inactive on FX. This is the first documentation of FVII and FXII activation for any *Echis* venom. The significant ontogenetic toxicological variations in *Echis* species were shown to impact antivenom efficacy. Among the tested antivenoms, SAVP-*Echis* was the most effective against both venom phenotypes, with adult venom being better neutralized. These findings suggest the need for a reconsideration of venom mixture selection in antivenom production through the inclusion of neonate venom. Additionally, the results indicate differential ontogenetic predatory ecology, providing a foundation for future natural history investigations.

## 1. Introduction

Snake envenomations lead to hundreds of thousands of people killed or permanently disabled annually, but these numbers are well-recognized as underestimations due to poor to non-existent reporting in some of the most affected regions [1,2,3,4,5,6,7,8,9]. Bad outcomes in terms of fatality and avoidable functional deficits result from delays in care most likely to be observed in underserved, low-resource areas [3,5,8,9,10]. Due to the lack of adequate and appropriate medical care, these snake bites have disproportionate impacts on the social and economic sides of these populations. Communities in Northern Africa, South America, and Southeast Asia are most heavily impacted by snakebite envenomations [5]. The viper genus *Echis* is responsible for some of the greatest global morbidity and mortality caused by venomous snakes and is a World Health Organization Category 1 species [11,12,13,14].

Currently, antivenoms are the only treatment option approved for envenomation, and areas with poor medical funding are often left with severe issues due to antivenoms’ short shelf life and high cost, leaving many facilities without appropriate treatments [12]. Venom biochemistry may vary dramatically due to geographical variations or shifts in diet through different life stages of snakes, leading to differential expression of toxin gene isoforms, with these variations ultimately impacting antivenom efficacy due to differential recognition by the antivenom IgG antibodies [15,16,17,18,19,20,21,22,23,24,25,26,27,28,29,30,31,32]. A spectacular example is the venom variation between *Pseudocerastes* species, with *Pseudocerastes fieldi* venom being powerfully neurotoxic [33,34,35,36,37] but *Pseudocerastes urarachnoides* venom being potently procoagulant through the activation of Factor X and prothrombin [38]. These differences in venom biochemistry that compromise the efficacy of antivenoms lead to an increased probability of devastating clinical manifestations even if patients reach appropriate care and nominally matched antivenoms [17,21,22,23,29,31,32].

Antivenom efficacy can vary even between closely related taxa that have the same venom biochemistry but vary in amino acids on key antigenic sites. The genus *Trimeresurus* underscores the fundamental consideration that phylogeny is a poor predictor of potency or antivenom efficacy. In this genus, the species with the most potent pathophysiological action of cleaving fibrinogen to form weak, transient fibrin clots in a pseudo-procoagulant manner (aka thrombin-like) are not each other’s closest relatives, but with amplification of this trait occurring on multiple convergent occasions within the genus [39]. Paralleling this, antivenom efficacy also does not follow phylogeny. In this case, the best-neutralized species (*T. albolabris*) is sister to the weakest antivenom neutralized species, while the next-best-neutralized species is a distant relative to it [39]. Another example is the destructive cleavage of fibrinogen by venoms from the genus *Causus*, with the venom of the long-glanded species *C. maculatus* recognized by the South African Venom Producer (SAVP) polyvalent antivenom, but the venom of the short-glanded species *C. lichtensteinii* was not neutralized [40]. Such examples are not limited to coagulotoxic venoms, such as *Crotalus scutulatus* subspecies which share the presynaptic neurotoxicity but are differentially neutralized by antivenom [41].

In other cases, the variation in antivenom efficacy is due to fundamental differences in the underlying biochemistry. For example, previous work demonstrated regional variation in the ability of *Bothrops atrox* venoms from adults to activate FX versus FII (prothrombin), which was shown to impact antivenom efficacy as the immunizing mixture was made using venoms rich in FII (prothrombin)-activating toxins, while containing less of FX-activating toxins, leading to populations with venom rich in FX-activating toxins being neutralized poorly [42]. Thus, even though all these *B. atrox* population variants had a net overall procoagulant mode of action, different biochemical tools driving the actions led to divergent antivenom efficacy.

Venom variations extend to age-related changes in venom biochemistry during the lifetime of the snake; for example, *Daboia russelii* varies in diet between neonates and adults (amphibian-dominated versus mammalian-dominated) and the venom varies accordingly, with the neonate phenotype more potent on amphibian plasma than adults [43]. Age-related venom variation may be problematic to treat if there is a shift in human pathophysiological effects. For example, the *Crotalus culminates* adult venom phenotype is anticoagulant, but the neonate venom phenotype is procoagulant [44]. As anticoagulant *Crotalus* venoms are used in the immunizing mixtures of the regionally available antivenoms, the procoagulant neonate venom is not neutralized, even by antivenoms that include venoms the procoagulant (but distantly related) *Bothrops* genus [44]. Another example is the Australian brown snake species (*Pseudonaja* spp.), whereby juveniles are nocturnal specialists that feed upon lizards and produce venom dominated by neurotoxic peptides (three-finger toxins, 3FTx), whereas adults are diurnal specialists that feed upon mammals and produce venoms dominated by procoagulant large proteins (Factor Xa enzyme and FVa protein) [45,46]. Consequently, neurotoxic envenomations by neonate snakes may be untreatable by antivenoms made using the adult venom coagulotoxic phenotype in the immunizing mixture.

The genus *Echis* consists of 4 main species clades, the *Echis pyramidum* clade, *E. coloratus* clade, *E. ocellatus* clade, and *E. carinatus* clade, containing at least 9 accepted species [47,48]. The venoms of all *Echis* species are potently procoagulant [29,49]. The coagulopathic clinical manifestations are due to the presence of snake venom metalloproteinases (SVMP) [50,51,52,53,54]. The procoagulant mechanism has been stated as due to the activation of Factor X and, to a lesser degree, prothrombin (with the prothrombin activation trait absent in some *Echis* venoms) [55,56,57,58,59,60,61,62,63,64,65,66,67,68,69].

*Echis* species have medical significance and have a vast impact across social and economic domains [19,29,53,54,70]. As *Echis* envenomations comprise a significant portion of global snake bites, venom variation within this genus presents a significant problem with regard to antivenom efficacy [17,21,22,31,32,71]. Intraspecific and interspecific variation in *Echis* have been attributed to varying diet and prey specificity [19,50,51]. This variation in venom significantly decreases antivenom cross-reactivity [29,50,51]. Ultimately, the combination of its distribution in heavily inhabited regions, venom variation, severe clinical pathology, and lack of cross-neutralization in antivenom culminates in *Echis* being dangerous with a very widespread social and economic impact throughout Northern Africa and Southern Asia [11,13,17,19,29,31,32,49,50,51,53,71,72,73]. While prey selective effects have been investigated for *Echis* venom [19], ontogenetic variations in the impact upon human plasma have not been investigated for this medically important genus. Therefore, the aim of this study was to elucidate if *E. p. pyramidum* presents an ontogenetic shift in its venom effects upon the human coagulation system and how this affects antivenom efficacy.

## 2. Results and Discussion

**Figure 1:**
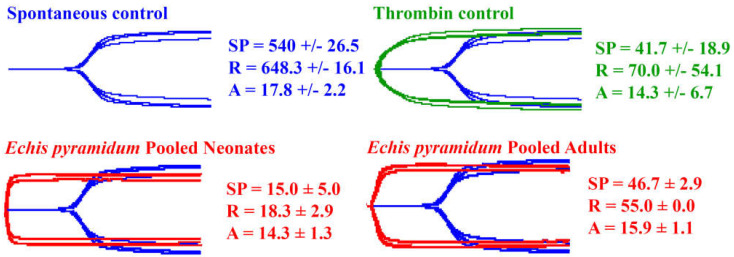
Thromboelastography on human plasma. Three clotting parameters (mean ± standard deviation, n = 4) are presented: split point (SP—time (seconds) until trace splits, representing clot initiation), reaction time (R—time (seconds) until amplitude = 2 mm, representing time until detectable clot), and amplitude (A—width of tracing at latest time point, representing clot strength). For easy visualization of venom effects, the spontaneous clotting control traces (blue) are included in the background of the venom effects traces (red). Data are n = 4 mean ± standard deviation.

Thromboelastography revealed all the venoms to be procoagulant, accelerating the clotting relative to the spontaneous clotting control and forming strong, stable fibrin clots (Figure 1). This is consistent with the activation of clotting factors, leading to the generation of endogenous thrombin and the subsequent conversion of fibrinogen to fibrin.

A Stago STA-R Max hemostasis analyzer was used to access differences in clotting time between pooled venoms. Relative to the spontaneous clotting control (444.10 ± 1.31 s), both venom phenotypes significantly accelerated clotting times (*p* < 0.0001), further indicating that these venoms are procoagulant. At the maximum venom concentration tested (20 µg/mL), there was a significant difference in neonate and adult plasma clotting times (*p* < 0.0001), with adult venoms clotting at 55.37 ± 1.19 s and neonates clotting at 11.33 ± 0.25 s (Figure 1). The procoagulant potency of each venom across all concentrations tested was assessed with area under the curve (AUC). Neonate venoms were 608.62 ± 2.32% more potent than adult venom. These results underscore that there is large ontogenetic venom variation in *E. pyramidum*. As such, while the neonates inject comparably less venom than adults, this could potentially be offset by the higher relative potency. As such, envenomations by neonates may be capable of producing the same or similarly severe clinical effect as bites by a larger adult snake.

**Figure 2:**
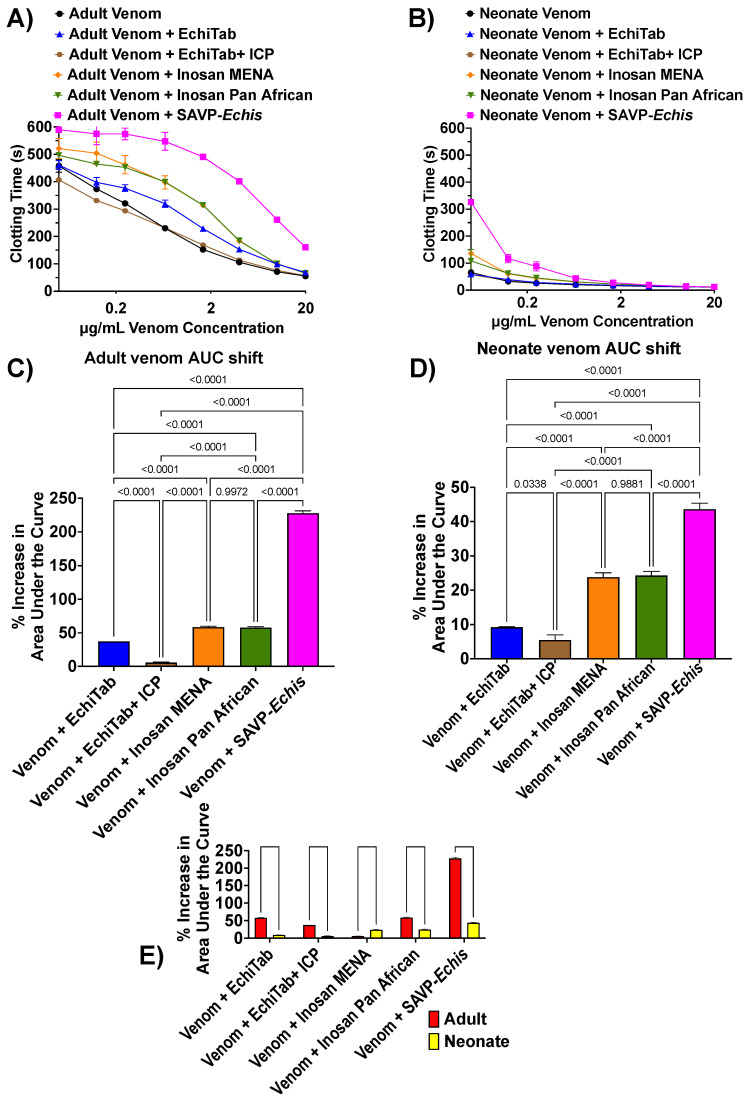
Logarithmic views of (**A**) adult and (**B**) neonate venom and antivenom human plasma clotting dose–response curves. (**C**) Adult and (**D**) neonate relative shifts in the area under the curve (AUC) for the venom and antivenom plasma clotting dose–response curves. No antivenom effect = 0%. *p*-values are from Brown–Forsythe and Welch ANOVA tests with post-hoc Dunnett’s T3 multiple comparisons. (**E**) Statistical comparisons between adult and neonate venoms for each antivenom type. *p*-values are from unpaired *t*-tests with Welch correction. Data are n = 3 mean ± standard deviation.

Previous work comparing SAVP-*Echis* and ICP+ Echitab antivenom demonstrated a strong response for SAVP-*Echis* against *E. pyramidum,* while ICP+ Echitab had little effect, but the Inosan antivenoms and Echitab were not included in the comparisons of that study [29]. As such, in this study, we expanded upon this previous work by including additional antivenoms and an ontogenetic venom comparison. Our data showed that the two venom phenotypes displayed a similar pattern of antivenom response relative to each other: SAVP-*Echis* > Inosan MENA = Inosan Pan Africa > Echitab > Echitab+ ICP (Figure 2A–D). In this study, there were significant differences between the two venom phenotypes, with the adult venoms neutralized more than the neonate, except for Inosan MENA (Figure 2E). For both venom phenotypes, the pattern of neutralization was consistent with SAVP-*Echis* and the Inosan antivenoms including *E. p. pyramidum* in their neutralizing mixtures, while the Echitab antivenoms only included *E. ocellatus* (Table 1). This is consistent with previous work showing the low recognition of ICP+ Echitab antivenom against *Echis* venoms from East Africa due to the inclusion in the immunization mixture of venoms only from West Africa [29].

**Figure 3:**
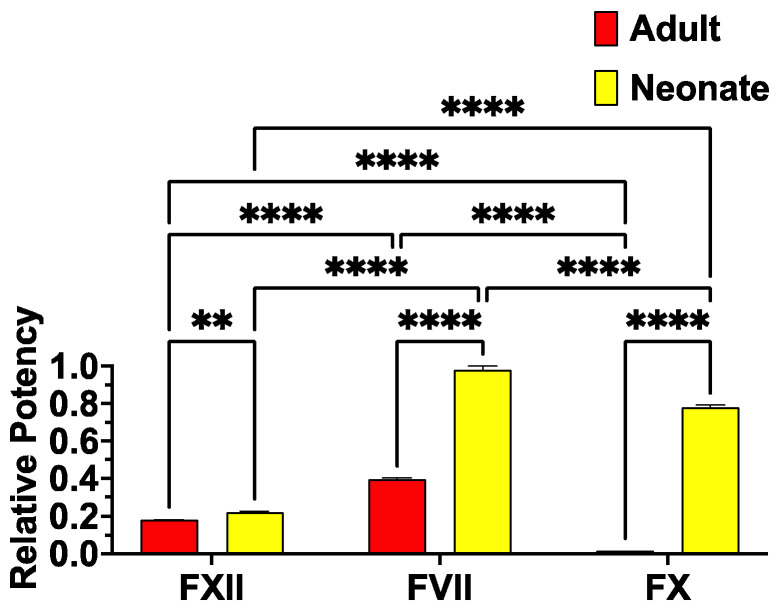
Adult and neonate relative ability to convert clotting zymogens of FXII, FVII, and FX into their corresponding activated enzymes. Statistics are Brown–Forsythe and Welch ANOVA tests with post-hoc Dunnett’s T3 multiple comparisons. *p*-values are comparisons between neonate and adult venoms within the same factor type, comparisons between factor types for neonate venom, and comparisons between factor types for adults. Data are n = 3 mean ± standard deviation. **** = *p* ≤ 0.0001, ** = *p* ≤ 0.01.

Neither venom phenotype activated FIX, FXI, or FII (prothrombin), but both venom sets activated FV, FX, and FXII. However, congruent with the differences in procoagulant activity, the two venom phenotypes differed in their ability to convert clotting Factors XII, VII, and X into the activated enzyme forms (Figure 3). The lack of activity on prothrombin is consistent with work showing that *Echis* venoms are not strong activators of this clotting factor, and that this trait is entirely absent in some populations [55,56,64,68]. It should be noted that a previous study which reported activation of prothrombin by *E. pyramidum* venom [68] did not report which subspecies was studied (*E. p. pyramidum* or *E. p. leakeyi*) or the locality of the venom. Therefore, regional variations in prothrombin activation within this species appear to be a significant variable worth investigating in future studies. In this study, while prothrombin activation was not a trait, both venom phenotypes were able to activate FXII, FVII, and FX. The neonate was significantly more potent for all three relative to the adult; however, the relative rank order differed between the two venom phenotypes. The rank orders were FVII > FXII >>> FX (negligible activity) for the adult venom phenotype and FVII > FX > FXII for the neonate venom phenotype. The differences in clotting factor activation paralleled the differences in antivenom neutralization (Figure 2E). This is consistent with all the antivenoms made using the adult *E. pyramidum* in the immunizing mixture. While other *Echis* species in the immunizing mixture contain FX-activating toxins, there may be limited cross-reactivity. This hypothesis is consistent with the limited cross-reactivity demonstrated between species for *Echis* antivenoms [29]. However, this requires future testing for validation. Regardless, these results underscore the importance of testing ontogenetic venom variations since full neutralization may require the addition of neonate venoms into the immunizing mixture due to the presence of toxins that activate FX which are not present in the adult venom, along with the higher concentration of FVII-activating toxins in the neonate venom phenotype.

Both FVII and FXII have only been described infrequently before in reptile venoms. FVII activation has been documented for the true vipers *Macrovipera lebetina obtusa* [74] and *Vipera ammodytes meridionalis* [75], the Central American pit viper *Porthidium volcanicum* [76], the Australian elapid snakes [45,77,78], and for the natricid snake *Rhabdophis subminiatus* [79]. However, the toxin types responsible are divergent. Metalloproteinasesare used in *Echis*, *Macrovipera*, *Porthidium*, *Rhabdophis*, and *Vipera* venoms. In contrast, the Australian elapids use weaponized forms of the blood clotting enzyme FXa. Therefore, FVII activation has convergently evolved at least two times within snake venom (once using SVMP, another time using FXa). However, FVII activation by SVMP in snake venoms may be a neofunctionalized trait, evolving on more than one occasion in some combination of *Echis*, *Macrovipera*, *Porthidium*, *Rhabdophis*, and *Vipera* venoms. These genera do not form a monophyletic procoagulant clade within snakes, but FVII activation may be a basal trait of true vipers, and emerging convergently two additional times (*Porthidium* and *Rhabdophis*). Reconstruction of the molecular evolutionary history of the toxins will be necessary to determine the number of times this trait has evolved in snakes. Regardless of the evolutionary patterns, FVII activation has clearly convergently evolved within lizard venoms, having been described for *Heloderma* venoms (with the toxin class responsible yet to be ascertained) [80]. In addition to activating FVII, snake venom metalloproteinases in *P. volcanicum* and *R. subminiatus* were previously also shown to activate FXII, again likely representing two separate neofunctionalization events within the SVMP toxin type [76,79]. Again, this trait has also convergently evolved within reptile venoms, having been described for *Heloderma* venoms (with the toxin class responsible again yet to be ascertained) [80].

Further research should be undertaken to determine the ecological and evolutionary drivers of the observed *E. p. pyramidum* ontogenetic venom variation in effects upon the vertebrate blood clotting cascade. A particular strength of this study was that it was performed on human plasma samples. The effects of plasma with platelets or whole blood were not assessed but could yield valuable insights in future studies. Other species within *Echis* should also be investigated for ontogenetic shifts in venom phenotype relative to the vertebrate blood system. In addition, ontogenetic variations in invertebrate clotting systems would be of interest in light of the large amounts of arthropods consumed by young *Echis* specimens [19].

Future research investigating proteomics and transcriptomics may shed light on the ontogenetic SVMP isoform variations that produce the fascinating variations in procoagulant potency and specificity. While both adult and neonate venoms were procoagulant, they differed in the underlying biochemistry, with impacts upon antivenom efficacy.

The wide distribution of *Echis* and the social and economic impact they cause make them a particularly important genus to study. This research should also be considered with its relevance to the development of antivenoms and small molecule therapeutics under development. These data suggest that the neonate venom should be included in the immunizing samples used to generate the antivenoms. As such, the new data presented in this paper have implications for the evidence-based design of clinically useful therapeutics.

## 3. Materials and Methods

### 3.1. Venom

All venom work was performed under the University of Queensland Animal Ethics Approval 2021/AE000075 and UQ Biosafety Committee Approval # IBC/134B/SBS/2015. Six lyophilized *Echis pyramidum pyramidum* venoms were provided by alpha-biotoxins. Samples included venom from two adult individuals (male and female from Egypt) and their offspring (four neonates—milked at 1 month old). Venom samples were stored at −80 °C before use. Venom was reconstituted with double deionized water (ddH_2_O), then centrifuged at 14,000 RCF at 4 °C for 10 min. The supernatant was aliquoted into a separate tube and its protein concentration was determined using the Thermo Fisher Scientific™ NanoDrop 2000 (Sydney, Australia) measuring absorbance at 280 nm. Due to the very small yields, venoms were then pooled into adult and neonate venom sets to allow for sufficient stocks for assays. Each venom set was prepared into a final stock solution of 1 mg/mL in 1:1 ddH_2_O: glycerol and kept at −20 °C until needed.

### 3.2. Plasma

Human plasma work was performed under University of Queensland Biosafety Approval #IBC134BSBS2015 and Human Ethics Approval #2016000256. Australian Red Cross (44 Musk Street, Kelvin Grove, QLD 4059, Australia) supplied human platelet-poor plasma (3.2% citrated) under research approval #16- 04QLD-10. Plasma was aliquoted by defrosting in a 37 °C water bath, aliquoted into 1.5 mL tubes, flash frozen in liquid nitrogen, and stored at −80 °C until required. For testing, plasma aliquots were defrosted in a 37 °C water bath for 5 min before use.

### 3.3. Thromboelastography (TEG)

Two TEG^®^ 5000 Thrombelastograph^®^ Haemostasis Analyser systems (Sydney, Australia) were used to test the effect of neonate and adult E. p. pyramidum venom on clot formation. For each test, ‘TEG^®^ 5000 disposable cups and pins clear’ (Haemonetics^®^, REF 6211) were loaded onto the machine. In each cup, 7 μL of 1 mg/mL venom stock was mixed with 72 μL of 0.025 M CaCl_2,_ 72 μL of phospholipid (dissolved in OK buffer), and 20 μL of Ok buffer. An amount of 189 μL of human plasma was then added to the cup, the sample pipette was mixed, and then immediately ran for 30 min at 37 °C. For negative controls, ddH_2_O:Glycerol replaced the venom sample. For positive controls, thrombin (Stago Liquid Fib kit cat. #00673) replaced the venom sample. The assay was based on previously conducted testing following the same methodology [39,74].

### 3.4. Coagulation Testing

Coagulation testing was conducted using a Stago STA-R Max hemostasis analyzer (Stago, Asnières sur Seine, France) and following previously established assays [39,74,78]. The clotting time of venoms was recorded in triplicate. First, 0.1 mg/mL venom samples (diluted with OK buffer (Stago Cat# 00360)) were loaded into the machine where they were further diluted (20, 10, 4, 1.66, 0.66, 0.25, 0.125, and 0.05 μg/mL). Then, 50 μL of each respective venom dilution, 50 μL 0.025 M CaCl_2_ (Stago Cat# 00367), 50 μL phospholipid (Stago Cat# 00597, dissolved in OK buffer), and 25 μL OK buffer were incubated together for 120 s. Then, 75 μL of plasma was added, and clotting was measured by the machine. The negative control (spontaneous control) substituted venom with 1:1 milliQ:Glycerol at 0.1 mg/mL in OK buffer. The positive control incubated 50 μL of Kaolin, 50 μL phospholipid, 25 μL OK buffer, and 75 μL of plasma for 120 s, and then 50 μL CaCl_2_ (25 mM) was added, and clotting was measured. The machine tested 8 concentrations of venom to generate a venom dilution curve. Positive and negative controls were analyzed in triplicate at 20 μg/mL to ensure healthy plasma and reagents were used.

### 3.5. Antivenom Neutralization Studies

Antivenoms were reconstituted in a solvent according to the manufacturer’s directions, then centrifuged at 14,000 RCF at 4 °C for 10 min, before being aliquoted into 2 mL tubes and stored at 4 °C. Antivenom samples were made to 5% (dissolved in OK buffer) and loaded into the Stago STA-R Max hemostasis analyzer. Antivenom assays followed the same protocol as the coagulation assay, except 25 μL OK buffer was substituted for 25 μL antivenom. Negative controls used 1:1 ddH_2_O:glycerol instead of venom to ensure the antivenoms were not having abnormal effects on the plasma.

### 3.6. Factor Activation Studies

Zymogen activation (Factors VII, IX, X, XI, XII, and II (prothrombin activation)) was undertaken using the same reaction stoichiometry and reaction conditions as per [77,81]:

- Reagents were manually plated in 384-well plates (black, lot#1171125, Nunc™ Thermo Scientific, Rochester, NY, USA).
- Loaded into a Fluoroskan Ascent™ (Thermo Scientific, Vantaa, Finland), followed by automated pipetting of 70 μL of buffer containing 5 mM CaCl_2_, 150 mM NaCl, and 50 mM Tris-HCl (pH 7.3) and Fluorogenic Peptide Substrate (ES011Boc-Val-Pro-Arg-AMC. Boc: t-Butyloxycarbonyl; 7-Amino-4-methylcoumarin; R & D systems, Cat# ES011, Minneapolis, Minnesota) in 500:1 ratio to start the reaction.
- The plate was warmed up at 37 °C.
- The plate was shaken for 3 s before each measurement.
- The reaction was carried out 300 times at 390/460 nm (excitation/emission), and every 10 s, the fluorescence generated by the cleavage of the substrate was measured by Ascent^®^ Software v2.6 (Thermo Scientific, Vantaa, Finland).
- To obtain final results, subtraction of “venom without zymogen” values from “venom with zymogen” values was calculated to nullify artificial increment of the fluorescence values caused by some venoms that work directly on the substrate.
- Finally, the resultant values from the subtractions were normalized as a percentage relative to the activated factors/enzyme (kaolin in case of FXII) by organizing in Excel and then analyzing in GraphPad PRISM 8.1.1 (GraphPad Prism Inc., La Jolla, CA, USA).

### 3.7. Statistical Analysis

Analyses were performed in GraphPad Prism 9.5.1 with *p*-values of less than 0.05 deemed statistically significant. Statistical tests used are presented in the figure captions. GraphPad Prism 9.5.1 was used to determine the area under the curve (*AUC*) and thus to calculate the % shift using the formula

$$(\frac{Venom\;+\;Antivenom\;AUC}{Venom\;AUC}-1)*100$$

A value of 0 indicates that the antivenom exhibited no neutralization whilst values above 0 demonstrated that there was neutralization.

## Figures and Tables

**Table 1:** Antivenoms used for testing.

Antivenom	Company	Country of Production	Species Used in Immunizing Mixture
**EchiTabPlus (*ET-Plus*)**	ICP	Costa Rica	*Bitis arietans*, *Echis ocellatus*, *Naja nigricollis*
**SAVP-*Echis***	SAVP (formerly SAIMR)	South Africa	*Echis ocellatus* and *E. pyramidum*
**EchiTabG (*ET-G*)**	MicroPharm	UK	*Echis ocellatus*
**Inoserp Pan-Africa (*Inoserp-P*)**	Inosan	Mexico, Spain	*Echis ocellatus*, *E. leucoaster*, *E. pyramidum*, *Bitis arietans*, *B. nasicornis*, *B. gabonica*, *Dendroaspus polylepis*, *D. viridis*, *D. angusticeps*, *D. jamesoni*, *Naja niricollis*, *N. melanoleuca*, *Naja haje*, and *Naja pallida.*
**Inoserp MENA**	Inosan	Mexico, Spain	*Bitis arietans*, *Cerastes cerastes*, *C. gasperettii*, *Daboia deserti*, *D. mauritanica*, *D. palestinae*, *Echis carinatus sochureki*, *E. coloratus*, *E. leucogaster*, *E. megalocephalus*, *E. pyramidum*, *Macrovipera lebetina obtusa*, *M. lebetina turanica*, *Montivipera bornmuelleri*, *M. raddei kurdistanica*, *Pseudocerastes persicus*, *Vipera latastei*, *Naja haje*, *N. nubiae*, *N. pallida*, and *Walterinnesia aeyptia.*

## Data Availability

The original contributions presented in this study are included in this article. Further inquiries can be directed to the corresponding author.
